# Validation of two short questionnaires assessing physical activity in colorectal cancer patients

**DOI:** 10.1186/s13102-018-0096-2

**Published:** 2018-05-29

**Authors:** Hege Berg Henriksen, Sveinung Berntsen, Ingvild Paur, Manuela Zucknick, Anne Juul Skjetne, Siv Kjølsrud Bøhn, Christine Henriksen, Sigbjørn Smeland, Monica Hauger Carlsen, Rune Blomhoff

**Affiliations:** 1 Department of Nutrition, Institute of Basic Medical Sciences, University of Oslo, Oslo, Norway; 2 Faculty of Health and Sports Science, University of Agder, Kristiansand, Norway; 3 Norwegian Advisory Unit on Disease-Related Malnutrition, Division of Cancer Medicine, Oslo University Hospital, Oslo, Norway; 4 Department of Biostatistics, Oslo Centre for Biostatistics and Epidemiology, Institute of Basic Medical Sciences, University of Oslo, Oslo, Norway; 5 Department for Clinical Service, Division of Cancer Medicine, Oslo University Hospital, Oslo, Norway; 6 Institute of Clinical Medicine University of Oslo, Oslo, Norway

**Keywords:** Short questionnaire, Physical activity, Sedentary time, SenseWear armband mini, Physical activity recommendations

## Abstract

**Background:**

In order to investigate the impact of adherence to recommendations of physical activity and sedentary time on health outcomes in clinical trials, there is a need for feasible tools such as questionnaires that can give representative estimates of these measures. The primary aim of the present study was to validate two such questionnaires and their ability to estimate adherence to the recommendations of physical activity defined as moderate-to- vigorous physical activity or moderate physical activity of at least 150 min/week in colorectal cancer patients. Secondarily, self-reported sedentary time from the HUNT-PAQ was also evaluated.

**Methods:**

Participants from 'The Norwegian dietary guidelines and colorectal cancer survival-study’ (CRC-NORDIET study) completed two short questionnaires; the NORDIET-FFQ (*n* = 78) and the HUNT-PAQ (*n* = 77). The physical activity monitor SenseWear Armband Mini was used as the reference method during seven consecutive days.

**Results:**

The NORDIET-FFQ provided better estimates of time in moderate-to- vigorous physical activity and moderate physical activity than the HUNT-PAQ. The NORDIET-FFQ was unable to rank individual time in moderate-to- vigorous physical activity and moderate physical activity (Spearman’s rho = 0.08, *p* = 0.509 and Spearman’s rho rho = 0.01, *p* = 0.402, respectively). All intensities were under-reported by the HUNT-PAQ, but ranking of individual time in moderate physical activity and sedentary time were acceptable among women only (Spearman’s rho = 0.37, *p* = 0.027 and Spearman’s rho = 0.36, *p* = 0.035, respectively). The HUNT-PAQ correctly classified 71% of those not meeting the recommendations (sensitivity), and the NORDIET-FFQ correctly classified 63% of those who met the recommendations (specificity). About 67% and 33% reported to meet the recommendation of moderate-to- vigorous physical activity with the NORDIET-FFQ and HUNT-PAQ, respectively, whereas 55% actually met the moderate-to- vigorous physical activity according to the SenseWear Armband Mini.

**Conclusions:**

The NORDIET-FFQ provided better specificity and better estimates of PA than the HUNT-PAQ. The HUNT-PAQ provided better sensitivity, and provided better ranking of PA and sedentary time among women than NORDIET-FFQ. It is important to be aware of the limitations documented in the present study.

**Trial registration:**

The study is registered on the National Institutes of Health Clinical Trials (Identifier: NCT01570010). Registered 4 April 2012.

**Electronic supplementary material:**

The online version of this article (10.1186/s13102-018-0096-2) contains supplementary material, which is available to authorized users.

## Background

The preventive effect of physical activity (PA) on risk of colorectal cancer is well-established [1–3]. However, an increasing number of studies also examine beneficial effects of PA during cancer treatment as well as in the posttreatment period [4–16], such as decreased all-cause mortality, increased disease-free survival, improved physical function and quality of life [5, 6, 11–13, 17, 18]. Moreover, reduced sedentary time, such as sitting during daytime, may be associated with reduced mortality and lower risk of recurrence in cancer patients [19–22].

The recommendations of PA for cancer patients and survivors provided by the American Cancer Society [23] emphasize that exercise is safe and feasible during cancer treatment, and improves outcomes such as physical function, fatigue and completion of chemotherapy [23]. The American Cancer Society, the World Health Organization and others [24–27] recommend at least 150 min of moderate intensity PA (MPA) or 75 min of vigorous intensity PA (VPA) per week or an equivalent combination. In 2011, the Norwegian Directorate of Health published the Norwegian Food-Based Dietary Guidelines (FBDG) which also includes similar recommendations on PA as well as for sedentary time [3].

In Norway, colorectal cancer (CRC) is the third most common cancer type, and the incidence is among the highest in Europe [28]. Implementing the recommendations of PA and incorporating specific exercises in the clinical care may improve the health outcomes of CRC patients [1–3, 24].

In order to estimate adherence to PA recommendations according to the Norwegian FBDG in a Norwegian CRC population, a valid and accurate physical assessment tool is needed. Importantly, assessment of adherence to the PA recommendations is required in counselling and when evaluating effectiveness of intervention studies. The use of objective monitors to record PA has increased during recent decades and gives valid and reliable data on intensity of PA and energy expenditure [29]. However, these activity monitors are expensive and time consuming for the clinician and researcher, particularly when recording PA in larger populations. Therefore, less expensive and easier methods are required to measure adherence to PA recommendations.

The most common self-reporting method to assess PA is the use of questionnaires [30, 31]. Over the past 2 or 3 decades, more than 30 PA questionnaires have been developed and validated [32]. Long questionnaires are challenging to complete for cancer patients often experiencing treatment and disease related side-effects such as fatigue and functional decline [33–36]. Questionnaires which contains few and well-defined questions regarding the different intensities of PA may be more suitable for this group of patients [37].

Although many previous questionnaires have been successfully used to assess PA, there is no questionnaire specifically designed to assess adherence to the PA recommendations as defined in Norwegian FBDG. For a clinical trial in colorectal cancer patients [38], a new short semi-quantitative questionnaire (NORDIET-FFQ) was developed to measure adherence to the Norwegian FBDG. The NORDIET-FFQ includes two questions on PA related to intensity levels similar to MPA and VPA. Another short questionnaire, the HUNT-PAQ [39] has been used in large healthy populations in Norway; however this questionnaire has not previously been validated in a CRC population.

Thus, the primary aim of the present study was to validate the two short questionnaires and their ability to estimate adherence to the PA recommendations according to the Norwegian FBDG. Secondarily, self-reported sedentary time from the HUNT-PAQ was also evaluated.

## Methods

### Subjects and study design

The present validation study was a sub-study of the ongoing CRC-NORDIET study, of which design and methods have been published elsewhere [38]. In brief, the aim of the CRC-NORDIET study is to investigate the effect of a diet similar to the Norwegian FBDG [3] on disease-free and overall survival among CRC patients post-diagnosis [38]. The risk factors shown to be related to CRC, i.e. diet and physical activity, are included in the Norwegian FBDG [40]. The CRC-NORDIET study is a prospective randomised controlled intervention trial, randomising 500 CRC patients into one of two study groups (i.e. 250 to diet intervention group and 250 to the control group). All patients are invited to the Study centre 3 times during the intensive 1-year intervention (i.e. at baseline 2–9 months post-surgery and at the two visits 6- and 12 months after baseline), and subsequently followed up for 14 years. Both study groups are offered equal recommendations on PA [38].

All patients from both study groups in the CRC-NORDIET study, who attended the follow-up at 6 months after baseline of intervention (i.e. 2–9 months post-surgery) from January 2014 to October 2015, were invited to take part in the present validation study. The patients were men and women aged 50–80 years old, with a confirmed CRC (ICD-10 C18–20), and staged I-III (i.e. locoregional disease without metastasis) according to the TNM staging system [41]. None of the patients included in the validation study underwent chemotherapy during the time-frame covered by the physical assessment methods used in the validation study (e.g. mean time from last chemotherapy injection to the start of the validation study (i.e 6 months after baseline) was 155 days among the 15% who received adjuvant treatment). During the 6-months visit, the patients completed the self-administered NORDIET-FFQ and HUNT-PAQ. In addition, they received the SenseWear Armband Mini (SWA), which was returned by mail to the CRC-NORDIET-study at the end of the test period of 7 days. Exclusion criteria for the present study were pacemaker implantation, not completed questionnaires or not wearing the SWA.

### Characteristics of the participants

To characterize the subjects, anthropometric measurements (weight, height, and hip-and waist circumference) and physical tests (hand-grip strength and 30-s sit-to-stand test) were measured by the researchers of the CRC-NORDIET study during all visits at the Study centre, as previously described elsewhere [38]. In addition, education level and smoking status were self-reported by completion of questionnaires during the visits at the Study centre. Information about tumour location status was retrieved from medical records in cooperation with the hospital personnel. Energy expenditure (kJ/d) was estimated from the SWA.

### Short semi-quantitative frequency questionnaire (NORDIET-FFQ)

The NORDIET-FFQ (available upon request to corresponding author), was designed to report both dietary intakes and PA in recent weeks (i.e. the last 1–2 months). The validity of the dietary items of the NORDIET-FFQ has been published elsewhere [42]. Completion of the NORDIET-FFQ as well as protocol for data handling followed the same procedures as described in detail in Henriksen et al. [42]. In brief, the NORDIET-FFQ was completed by the patients at the Study centre. The completed questionnaires were scanned by trained researchers and the image files translated into data files using the Cardiff Teleform 2006 Software (6.0) (Datascan). The last two questions in the NORDIET-FFQ asked for PA with two different intensities, MPA and VPA estimated in pre-defined intervals of frequency per week and duration in minutes. The explanatory texts for both PA questions included examples of typical activities: moderate intensity was exemplified by brisk walking, household chores or other activities resulting in slight breathlessness, and vigorous intensity was exemplified by running, cross-country skiing or other activities resulting in high breathlessness. The question about frequency contained different responses in times per week (time divided by seven) coded as follows: *0 = 0*, *1 = 0.14*, *2 = 0.29*, *3 = 0.43*, *4 = 0.57*, *5 = 0.71*, *6–7 = 0.93* and *8+ = 1.37* (added 20% to 8 and divided by 7). Moreover the responses of duration in minutes were coded as follows: *1–4 = 2*, *5–9 = 7*, *10–15 = 12.5*, *16–20 = 18*, *21–30 = 25.5*, *31–45 = 38*, *46–60 = 53* and *60 + =72* (added 20% to 60). Amounts in minutes of PA per day for each intensity were calculated by multiplying frequency (i.e. times per day) with duration (i.e. minutes each time). This resulted in variables of total-MPA and total-VPA, of which all minutes within each intensity were included (Additional files 1 and 2). Additionally, categories of 10-min bouts were computed and defined as ten or more consecutive minutes within each intensity level. This resulted in data on 10-min bouts of MPA and VPA.

### HUNT (the Nord-Trøndelag health study) physical activity questionnaire (HUNT-PAQ)

The HUNT-PAQ was based on the questionnaire used in the HUNT 3-study [39]. Only the five questions about PA as described in Kurtze et al. [43] were used. The question about frequency contained the following responses: *Never* and *Less than once a week,* both coded as 0, *Once a week* coded as 1, *2–3 times a week* coded as 2.5, and *Almost every day* coded as 7. The question about duration of activity contained the following responses: *Less than 15 min* coded as 12 (subtracted 20% from 15), *15–29* min coded as 22, *30–1 h* coded as 45 and *more than 1 h* coded as 72 (added 20% to 60). The products of frequency and duration were weighed by intensity level, i.e. *Low, Moderate* or *Vigorous* coded as 1, 2 and 3, respectively. The low intensity level was not evaluated in the present study. Additionally, there was a question about daily sedentary time in hours on a usual day (not included sleeping at night-time). The questionnaire generated data on activities in bouts of 10 and more consecutive minutes for each intensity level, such as MPA and VPA.

### Objective physical activity measurement

The objective PA monitor SenseWear Armband Mini (SWA) (BodyMedia, Pittsburgh, Pennsylvania, USA) was used to record daily PA and energy expenditure during seven consecutive days [44]. A priori, we defined a valid day of recording if the wear time was ≥80% of a 24-h sampling period. The SWA has previously been validated against double-labelled water [45], indirect calorimetry [46] and other accelerometers [47] in adults and cancer patients. It monitors physiological data such as heat flux, galvanic skin response, 3-axis accelerometer and skin temperature. The SWA was pre-programmed by the researcher with the co-predictors such as weight, height, birth date, sex, smoking status (smoker/non-smoker) and whether the participant was left or right handed, and placed around the triceps muscle halfway on the upper non-dominant arm. The participants were instructed to continue their normal activity level while wearing the SWA. Water-based activities were not recorded by the SWA because the monitor is not waterproof. Participants were asked to remove the SWA when performing activities in water. All data were retrieved from the SWA to a computer with the SenseWear Professional Software Version 7.0 BodyMedia Inc. (Pittsburgh, Pennsylvania, USA).

Activity intensities were integrated into algorithms, providing estimates of energy expenditure expressed in metabolic equivalents (METs). The definition of 1 MET is the amount of oxygen consumed while sitting at rest and is equal to 3.5 ml O_2_ per kg bodyweight per min [48]. Moderate and vigorous intensities were defined as 3–6 and > 6 METs, respectively, as calculated by Ainsworth and coworkers [49, 50]. Sedentary time was defined as all daily activities ≤1.5 METs, of which nighttime sleep was removed (a priori defined as from 12 midnight to 6.00 a.m.). All activities were calculated and expressed in minutes or hours per week (sedentary time).

The SWA records all intensities in 1-min intervals, which were translated into different categories of data such as total-MPA and total-VPA. Furthermore, the data were also computed into 10-min intervals, which were defined as ten or more consecutive minutes within the relevant intensity level. The bouts of 10-min were calculated for the two intensity levels, which gave data on bouts of 10-min for MPA and VPA.

### Recommendations of physical activity

The CRC patients were advice to follow the recommendations of moderate-to-vigorous intensity PA (MVPA), MPA and VPA. MVPA was defined as ‘MPA + (VPA*2)’ [51] and the cut-off points for fulfilling recommendations of MVPA and MPA were at least 150 min per week, and at least 75 min per week for VPA. The activities should be in bouts of 10 and more consecutive minutes.

### Statistical analysis

Statistical analyses were performed by use of IBM SPSS Statistics, version 22. Results were considered significant with two-sided *p*-values below 0.05. Normal distribution was checked for all data by inspection of histograms, normal Q-Q-Plots and Kolmogorov-Smirnov test (*p* > 0.05).

All anthropometric measurements and other characteristics of the study population were normally distributed and are presented as means with standard deviations (SD). The categorical data are presented as frequency with percentages and compared by the Fischer exact test and Pearson chi-square test. As most of the estimates of PA from the SWA, NORDIET-FFQ and HUNT-PAQ were not normally distributed, they are presented as medians and 5th - and 95th percentile. Statistical significant differences in median activity between the two questionnaires compared to the SWA were tested with Wilcoxon Signed-Rank test for paired data. Bland-Altman plots with limits of agreements were used to explore the differences between the measurements from the two methods (i.e. questionnaire minus SWA) plotted against the average of the two measurements, for each individual subject, as well as to identify outliers [52, 53]. Systematic under- or over-reporting was tested by linear regression with MVPA and MPA from SWA as the independent variable and the difference between the questionnaires and SWA as the dependent variable. Ranking of individual time in PA and degree of association between the continuous variables from the two different methods were analysed by Spearman Rank Order Correlation (rho). The ability of the NORDIET-FFQ and the HUNT-PAQ to classify the individual’s activity intensity into the same category as the SWA was estimated by the use of sensitivity and specificity analysis. Sensitivity was defined as the number of subjects reported not to fulfil the MVPA with both questionnaires and SWA as a percentage of those who had reported not to fulfil the MVPA with the SWA. Specificity was defined as the number of subjects reported to fulfil the MVPA with both questionnaires and SWA as a percentage of those who had reported to fulfil the MVPA with the SWA.

### Sample size

In order to detect a Pearson correlation coefficient of 0.5 or higher between the test-method (i.e. questionnaires) and the reference method (i.e. SWA), a sample size of 38 men and 38 women was required to achieve a significance level of 5% and power of 90% [54]. With an expected consent rate of 90% and exclusion rate of maximum 5%, we aimed to invite 90 participants in order to include 76 participants to the present study.

## Results

Of the 88 invited participants, three were excluded due do pacemaker implantation and 7 declined to participate. Hence, 78 participants used the SWA and completed the NORDIET-FFQ, whereas 77 of these also completed the HUNT-PAQ. General characteristics of the participants are presented in Table 1. Mean age of the participants was 64.8 years, and did not differ significantly between men and women (Table 1). Mean time between surgery and baseline was 120.7 days ±41.4 days (mean ± SD) and between baseline and 6-months visit was 184.4 ± 39.1 days (mean ± SD). Total energy expenditure estimated from the SWA was 11.4 and 9.0 MJ for men and women, respectively. About 8% of the participants were smokers and 51% were highly educated (college/university education) (Table 1).

**Table 1:** Characteristics of all participants in total and stratified by men and women (mean (SD))

Variables	Total (*n* = 78)	Men (*n* = 42)	Women (*n* = 36)	p^a^
Age, years, mean (SD)	64.8 (7)	65.2 (7.4)	64.3 (7.4)	0.590
Smokers, n (%)	6 (7.7%)	3 (7%)	3 (8%)	1.000
EE, kJ/d^b^, mean (SD)	10,378 (1909)	11,496 (1474)	9074 (1488)	< 0.001
Education, n (%) (total *n* = 78, men *n* = 42, women *n* = 36)				
Primary school	4 (5)	3 (7)	1 (3)	0.370
Lower secondary/High school	34 (44)	21 (50)	13 (36)	
College/University	40 (51)	18 (43)	22 (61)	
Anthropometry (mean, SD) (total *n* = 78, men *n* = 42, women *n* = 36)				
Weight, kg	79.2 (16.3)	87.0 (11.9)	70.0 (16.1)	< 0.001
Height, m	1.73 (8.31)	1.77 (6.7)	1.66 (5.3)	< 0.001
BMI, kg/m^2^	26.1 (5)	27.5 (3.7)	25.2 (5.5)	0.030
Waist circumference	93.9 (13.7)	100.7 (9.6)	86.2 (13.8)	< 0.001
Hip circumference	101.0 (9.2)	101.6 (6.9)	100.3 (11.4)	0.560
Tumor classification n (%) (total *n* = 70, men *n* = 36, women *n* = 34)				
TNM I	13 (17)	9 (25)	4 (12)	0.170
TNM II	33 (42)	18 (50)	15 (44)	
TNM III	24 (31)	9 (25)	15 (44)	
Physical performance (mean, SD) (total *n* = 78, men *n* = 42, women *n* = 36)				
Hand-grip strength right, kg^c^	34.3 (9.5)	40.9 (6.6)	26.7 (5.9)	< 0.001
Hand-grip strength left, kg^c^	31.1 (9.7)	37.9 (7.1)	23.5 (5.7)	< 0.001
Sit-to-stand test	17.3 (5.3)	17.9 (5.8)	16.5 (4.6)	0.220

*TNM* tumor node metastases, *BMI* body mass index, *EE* energy expenditure

^a^Continuously variables were tested with Student t-test. Categorical variables were tested by the Fischer exact test (two-sided)

^b^Estimated energy expenditure from the physical activity monitor SenseWear Armband Mini (BodyMedia, Pittsburgh, Pennsylvania, USA) (SWA)

^c^The maximal strength of hand grip (kg) was recorded. For women and men, a 40 kg- and 80 kg-spring was used, respectively

### Moderate-to-vigorous intensity physical activity recorded from the NORDIET-FFQ, HUNT- PAQ and SWA

Participants wore the SWA monitors for 97.9 ± 3.8% (mean, ± SD) of the time during 6.2 ± 0.8 days (mean, ± SD) of monitoring.

Median duration of PA estimated from the NORDIET-FFQ, HUNT-PAQ and the SWA is presented in Table 2. There was no significant difference between the NORDIET-FFQ and SWA for the measure for activity of moderate-to-vigorous intensity PA (MVPA), either for the total population (*p* = 0.897) or when analyzing sexes separately. The HUNT-PAQ, however, significantly measured MVPA differently on a group level compared to SWA (*p* < 0.001).

**Table 2:** Physical activities and sedentary time, all participants in total and stratified by sex

Physical activity (min/week)^a^	NORDIET-FFQ			SWA			NORDIET-FFQ/SWA *p*-values^b^		
	Total (*n* = 78)	Men (*n* = 42)	Women (*n* = 36)	Total (*n* = 78)	Men (*n* = 42)	Women (*n* = 36)	p_tot_	p_male_	p_female_
	Median (P_5_, P_95_)	Median (P_5_, P_95_)	Median (P_5_, P_95_)	Median (P_5_, P_95_)	Median (P_5_, P_95_)	Median (P_5_, P_95_)			
MVPA	247 (0,691)	226 (0,812)	247 (0,641)	187 (12,881)	200 (2,1342)	169 (24,615)	0.897	0.759	0.838
MPA	152 (0,469)	159 (0,469)	152 (0,469)	187 (12,691)	200 (2872)	169 (11,615)	0.007	0.050	0.090
VPA	0 (0,219)	0 (0,256)	0 (0,236)	0 (0,42)	0 (0,44)	0 (0,43)	< 0.001	0.011	0.005
	HUNT-PAQ			SWA			HUNT-PAQ/SWA *p*-values^b^		
	Total (*n* = 77)	Men (*n* = 42)	Women (*n* = 35)	Total (*n* = 77)	Men (*n* = 42)	Women (*n* = 35)	p_tot_	p_male_	p_female_
MVPA	72 (0,504)	38 (0,504)	113 (0,389)	182 (11,881)	200 (2,1342)	156 (23,618)	< 0.001	< 0.001	0.001
MPA	55 (0,504)	38 (0,504)	113 (0,353)	182 (11,696)	200 (2872)	156 (11,618)	< 0.001	< 0.001	0.002
Sedentary time (h/day)	6 (0,12)	6 (0,13)	7 (0,12)	13 (10,15)	13 (10,15)	13 (10,15)	< 0.001	< 0.001	< 0.001

*NORDIET-FFQ* Norwegian Dietary Guidelines Food Frequency Questionnaire, *HUNT-PAQ* HUNT Physical Activity Questionnaire, *SWA* SenseWear Armband, *MVPA* (moderate intensity physical activity 10 min bouts + (vigorous intensity physical activity 10 min bouts*2)); bouts of 10 min = sum of at least 10 consecutive minutes of activity and above, *MPA* moderate intensity physical activity in bouts of 10 min, *VPA* vigorous intensity physical activity in bouts of 10 min

^a^Physical activity levels based on Norwegian Food Based Dietary Guidelines

^b^Wilcoxon signed rank test, *p*-values for median physical activity from NORDIET-FFQ, HUNT-PAQ and SWA, both total and between sex

**Fig. 1:**
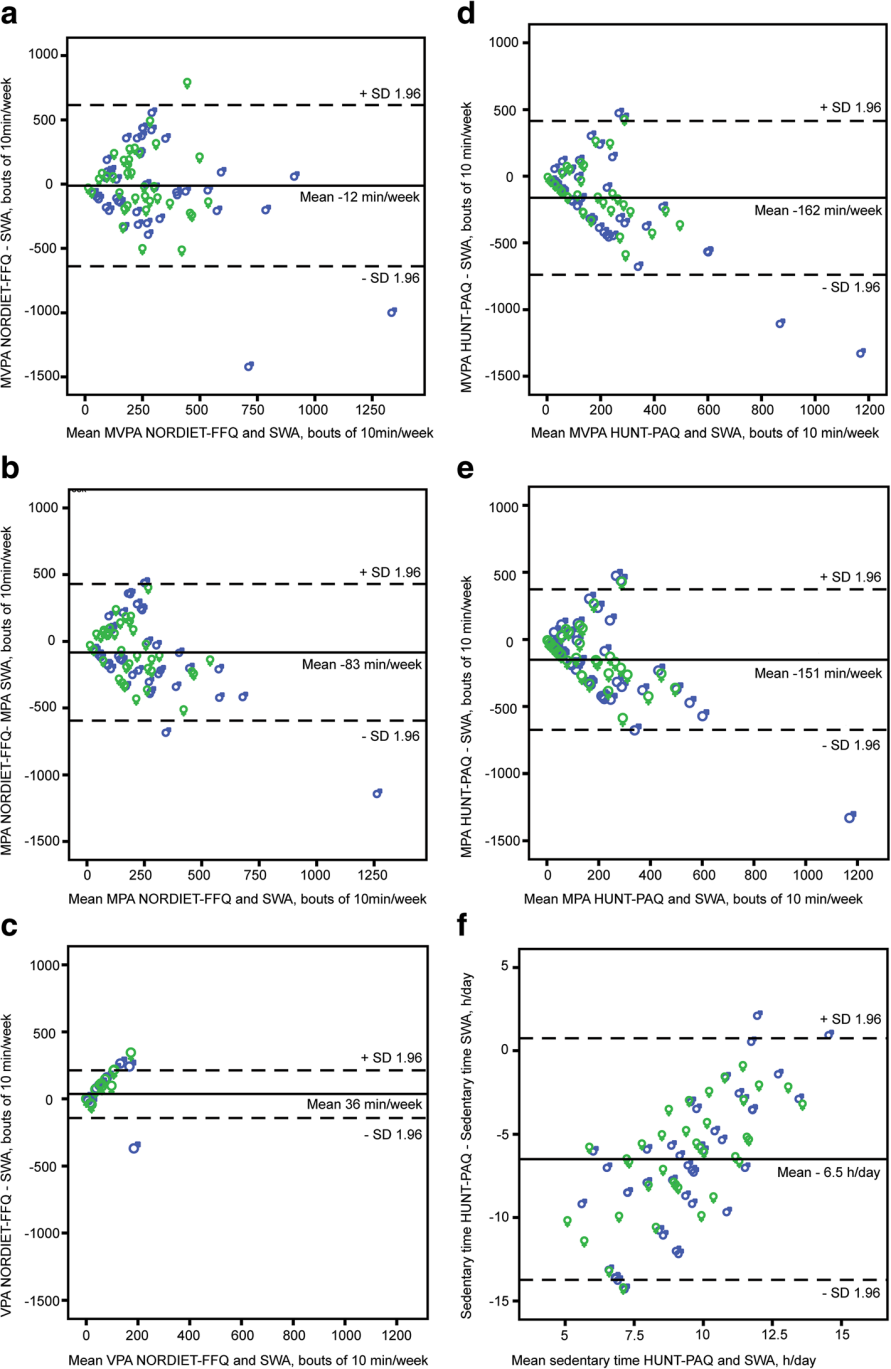
Bland-Altman plots depicting mean differences of the questionnaires minus SWA for physical activity; **a** MVPA minutes/week, NORDIET-FFQ, **b** moderate intensity physical activity in bouts of 10 min/week, NORDIET-FFQ; **c** vigorous intensity physical activity in bouts of 10 min per week, NORDIET-FFQ; **d** MVPA minutes per week, HUNT-PAQ; **e** moderate intensity physical activity in bouts of 10 min/week, HUNT-PAQ; **f** Sedentary time in hours/day, HUNT-PAQ. The solid line represents the mean, and the dashed lines represent the 1.96 SDs of the observations. Females denoted as ♀ and males denoted as ♂

Mean differences in PA measures (i.e. questionnaire minus SWA) with corresponding limits of agreements between questionnaires and the SWA are shown in Bland Altman plots in Fig. 1. MVPA (Fig. 1a) was reported only 4% differently with the NORDIET-FFQ compared to the SWA (mean difference and limits of agreement − 12 ± 624 min/week). The under-estimation of MVPA (Fig. 1d) with the HUNT-PAQ was 58% compared to SWA (mean difference and limits of agreement − 162 ± 576 min/week). Moreover, the Bland Altman-plot for MVPA revealed an increase in the differences between both questionnaires and SWA with increased PA level. Additionally, the differences were randomly and evenly distributed above and below the mean difference for both questionnaires compared to SWA up to about 250 min/week (Fig. 1a and d). The slope of the linear regression was negative and significant for both questionnaires (β = − 0.79, p < 0.001(NORDIET-FFQ) and β = − 0.85, p < 0.001 (HUNT-PAQ)), indicating under-reporting at higher levels of PA (MVPA from SWA as independent variable). Removing of two outliers shown in the Bland Altman plot (Fig. 1a) did not have any effect on the limits of agreement or the linear regression (data not shown). Therefore, they were included in further analyses. The Spearman’s rho of the MVPA was insignificant and weak (rho = 0.08, *p* = 0.509) for the NORDIET-FFQ, indicating that the questionnaire was not able to rank individual time in MVPA. Likewise, in the HUNT-PAQ, ranking of individual time in MVPA was also poor (Spearman’s rho of 0.14, *p* = 0.238). The NORDIET-FFQ captured 63% individuals fulfilling the recommendation of MVPA (specificity), whereas only 29% of those in need of PA counselling (sensitivity) (Table 3). The HUNT-PAQ was better at capturing individuals not fulfilling the recommendation of MVPA (sensitivity of 71%), but worse in identifying those who did (specificity of 36%).

**Table 3:** Sensitivity and specificity of the questionnaires in detecting adherence to the recommendations of physical activity

	Sensitivity n^a^ (%)		Specificity n^b^ (%)	
Physical activity intensity	NORDIET-FFQ	HUNT-PAQ	NORDIET-FFQ	HUNT-PAQ
MVPA> 150 min/week	10 (29)	25 (71)	27 (63)	15 (36)
MPA > 150 min/week	16 (46)	26 (74)	22 (51)	15 (36)
VPA > 75 min/week	57 (75)	–	0 (0)	0 (0)

*NORDIET-FFQ* NORDIET Food Frequency Questionnaire, *HUNT-PAQ* HUNT Physical Activity Questionnaire, *SWA* SenseWear Armband, MVPA = (moderate physical activity 10 min bouts + (vigorous physical activity 10 min bouts*2)); bouts = sum of at least 10 consecutive minutes of activity and above, *MPA* moderate physical activity in bouts of 10 min, *VPA* vigorous physical activity in bouts of 10 min

^a^subjects reported not fulfilling the recommendations for both the NORDIET-FFQ/HUNT-PAQ and SWA

^b^subjects reported fulfilling the recommendations for both the NORDIET-FFQ/HUNT-PAQ and SWA

### Moderate intensity physical activity recorded from the NORDIET-FFQ, HUNT- PAQ and SWA

Time spent in activity of moderate intensity PA (MPA) did not differ significantly by sex between the NORDIET-FFQ and SWA, whereas in HUNT-PAQ time in MPA was measured significantly different from SWA on group level (Table 2).

MPA was under-reported in both questionnaires as shown by the mean differences and limits of agreement from the Bland-Altman plots of − 83 ± 512 min/week and − 153 ± 523 min/week from the NORDIET-FFQ and HUNT-PAQ, respectively (Fig. 1b and e). The differences of MPA were randomly and evenly distributed above and below the mean difference for both questionnaires compared to SWA up to about 250 and 200 min/week with the NORDIET-FFQ and HUNT-PAQ, respectively (Fig. 1b and e). Linear regression also revealed a significant systematic under-reporting at higher levels of PA (MPA from SWA as independent variable) in both questionnaires (β = − 0.78, *p* < 0.001(NORDIET-FFQ) and β = − 0.83, p < 0.001 (HUNT-PAQ)).

Ranking of individual time in MPA was fair among women only (Spearman’s rho = 0.37, *p* = 0.027) with the HUNT-PAQ, but weak and insignificant with the NORDIET-FFQ (Spearman’s rho = 0.01, *p* = 0.402). The HUNT-PAQ captured 74% of individuals not fulfilling the recommendation of MPA (sensitivity), but only 36% of those who did (specificity). Both sensitivity and specificity for MPA were low with the NORDIET-FFQ (Table 3).

### Vigorous intensity physical activity recorded from the NORDIET-FFQ, HUNT- PAQ and SWA

Median time in activity at vigorous intensity PA (VPA) was reported significantly differently between both questionnaires and SWA. The Bland Altman plot for VPA revealed an over-reporting of 36 ± 176 min/week (mean difference ± limits of agreement) with the NORDIET-FFQ, which increased with increased activity (Fig. 1c). Since only one participant reported VPA with the HUNT-PAQ, data from this activity are not presented. Moreover, the NORDIET-FFQ identified 75% of the individuals not fulfilling VPA.

### Sedentary time recorded from the HUNT- PAQ and SWA

Amount of sedentary time was only measured in the HUNT-PAQ. Median time in sedentary intensity was significantly different between the questionnaire and SWA (Table 2). The Bland Altman plot revealed a high under-reporting of about 52% (6.5 h/day) compared to SWA, which decreased with increased sedentary time (Fig. 1f). However, the questionnaire was able to rank individuals according to sedentary time among women (*r* = 0.36. *p* = 0.035), but not among men or all participants in total.

### Adherence to the recommendations of physical activity recorded from the NORDIET-FFQ, HUNT- PAQ and SWA

Looking at each method separately, participants who reported to fulfil the MVPA of at least 150 min per week were 66% and 33% with the NORDIET-FFQ and HUNT-PAQ, respectively. However, only 55% of the participants actually met this recommendation according to the SWA (Table 4).

**Table 4:** Proportion of participants (n (%)) fulfilling the recommendations of physical activity with each measuring method

Recommendations of physical activity intensity	NORDIET-FFQ (*n* = 78)	HUNT-PAQ (*n* = 77)	SWA (*n* = 78)
MVPA > 150 min/week	52 (66.7%)	25 (32.5%)	43 (55.1%)
MPA > 150 min/week	41 (52.6%)	24 (31.2%)	43 (55.1%)

*NORDIET-FFQ* NORDIET Food Frequency Questionnaire, *HUNT-PAQ* HUNT Physical Activity Questionnaire, *SWA* SenseWear Armband Mini, MVPA = (moderate intensity physical activity 10 min bouts + (vigorous intensity physical activity 10 min bouts*2)); bouts = sum of at least 10 consecutive minutes of activity and above, *MPA* moderate intensity physical activity in bouts of 10 min, *VPA* vigorous intensity physical activity in bouts of 10 min

## Discussion

In the present study, we evaluated the ability of the questionnaires, NORDIET-FFQ and HUNT-PAQ, to estimate adherence to PA recommendations among CRC patients participating in the ongoing intervention, CRC-NORDIET study [38].

Generally, self-reported measures tend to over-report both duration and level of PA compared to objective methods [55], but under-reporting has also frequently been documented [55, 56] which may have several different explanations. A review of studies focusing on the comparison of objective measures versus self-reporting of PA was performed by Prince et al. [55]. They found that self-reported measures of PA were higher than the objective measure when accelerometers were used. However, in the present study MVPA (only HUNT-PAQ) and MPA were under-reported with the questionnaires compared to SWA. This may be for several reasons; firstly, the intensity level of MPA was defined as activities resulting in slight breathlessness. Cancer patients undergoing disease-related treatment and in a recovery phase post-surgery might experience breathlessness at lighter intensity than before, due to treatment effects and comorbidities such as anaemia, chronic obstructive pulmonary disease, and physical deconditioning [57, 58]. Breathlessness may result in over-reporting of higher intensity (VPA) and under-reporting of MPA. Slightly reduced physical function, measured by handgrip-strength and 30-s sit-to-stand test, was observed in the CRC patients participating in the present study as compared to healthy individuals in Norway (Table 1) [59].

Secondly, the under-reporting of MPA might also be explained by the different techniques in recording physical activities used by the two methods. All activities are recorded by the SWA within a 24 h day, whereas the questionnaires rely on the participant’s memory and subjective evaluation of activity while responding to just a few questions [60].

Thirdly, the degree of under-reporting of MVPA and MPA was higher with the HUNT-PAQ than with the NORDIET-FFQ. This might be due to the restricted opportunity for the participants to report both MPA and VPA in the HUNT-PAQ, which is possible with the NORDIET-FFQ. Moreover, under-reporting may also be explained by the different reporting intervals of frequencies in the responses; the NORDIET-FFQ contained responses for activities lasting both less than and above 10-min intervals, while the HUNT-PAQ only asked for activities lasting more than 10-min intervals. Therefore, increased accuracy in reporting of intensities was possible with the NORDIET-FFQ compared to the HUNT-PAQ, since intensities performed for less than 10 min were not recorded with the HUNT-PAQ.

Bias in reporting of intensity seems to be influenced by the amount of questions for a specific activity within a questionnaire, i.e. whether it contains a single-item question or domain-item questions [61–64]. The self-reported sedentary time in the present study was based on a single-item question and was greatly under-reported by the HUNT-PAQ compared to SWA, an effect supported by other studies [61, 64]. Since the HUNT-PAQ asked for sedentary time during day-time, a general definition of a day in the SWA was performed by removing night-hours between midnight and 6 am. Consequently, sedentary time during day-time recorded by the SWA was calculated from 6 am to midnight. However, this definition may be challenged in cancer patients facing several disease- and treatment side-effects influencing sleeping pattern due to increased need for resting time [65]. A diary report from each participant would probably improve the definition of night-time resulting in higher precision in reporting sedentary time during day-time.

Vassbakk- Brovold et al. [34] documented an over-reporting of 366% of MVPA recommendation with the short form International Physical Activity Questionnaire (IPAQ-sf) compared to the SWA among cancer patients undergoing chemotherapy. The IPAQ-sf contains 9 questions on PA [66], whereas the NORDIET-FFQ and HUNT-PAQ contains 2 and 4 questions on PA, respectively. Both questionnaires in the present study contained few detailed question about type of PA activities. Thus, under-reporting of the activities may be due to decreased precision in reporting different kinds of activities during a day. However, the number of questions depends on the rationale of the questionnaire. In the present study, the aim was to estimate adherence to the PA recommendations based on the Norwegian FBDG. In clinical practice as well as intervention studies, it is advantageous to have a short and easy PA assessment tool to be used when monitoring adherence to the PA recommendations.

A small mean difference of only 4% was revealed for MVPA by the NORDIET-FFQ, whereas HUNT-PAQ under-estimated by 58% compared to the SWA. This is comparable with previous studies, which have reported mean differences around 44% (ranging from − 78% to 500%) [55]. Evenly distributed differences above and below the mean difference in the Bland Altman plots indicated no systematic bias of activities in any of the questionnaires. However, linear regression revealed a systematic bias as shown by the significant negative slope for both questionnaires, indicating a trend towards more under-reporting with increased amount of PA. As can be seen from the Bland Altman plots, this negative trend seems to be accounted for by intensities higher than 250 and 200 min/week with the NORDIET-FFQ and HUNT-PAQ, respectively.

The limits of agreements were wide for both questionnaires, indicating weak ability to assess MVPA and MPA on an individual level. This has been supported by Ekelund et al. [67] and by Vassbakk- Brovold et al. [34], who validated the short form of the International Physical Activity Questionnaire (IPAQ-s) against an objective monitor among healthy Swedish adults and adult cancer patients, respectively. In the present study, limits of agreement were smaller at 150 min/week for MVPA and MPA for both questionnaires (about 500 min/week) than at higher levels of PA.

Hence, the NORDIET-FFQ was able to measure intensities up to about 250 min/week (i.e. including the PA recommendation of at least 150 min/week), but the HUNT-PAQ was less well suited to measure the corresponding intensities.

Studies including physical activities categorized in terms of different levels of exertion (light, moderate, vigorous) tend to result in more outliers, with VPA contributing the most outliers [55]. The present study reported more outliers at higher levels of all intensities, of which the more extreme differences in reporting tended to be among males. Importantly, there were few observations with high amounts of PA, indicating high uncertainty and low interpretation of those data.

Previous studies differ in degrees of correlation between self-reported methods and objective measurements of PA [55], with no specific trend. In the present study, there were poor correlations for all variables between the NORDIET-FFQ and SWA, whereas fair correlations were found between the HUNT-PAQ and SWA for MPA and sedentary time among women only. Ranking of individuals according to time in MPA and sedentary time were thus fairly good with the HUNT-PAQ.

NORDIET-FFQ identified 63% of individuals fulfilling the MVPA (specificity), but was not able to identify those in need of PA counselling (sensitivity). However, the HUNT-PAQ was able to identify 71% not fulfilling the MVPA and 36% of those who did. Hence, the NORDIET-FFQ provided a fairly specific measure of PA, but limited sensitivity to correctly classify individuals not fulfilling the MVPA. Thus, NORDIET-FFQ should be used with care in a clinical setting. In contrast, the HUNT-PAQ was able to identify those in need of PA counselling, but limited in identifying those who fulfilled the PA recommendations.

About 66% reported meeting the recommended level of MVPA with the NORDIET-FFQ (i.e. 150 min/week) whereas 55% actually met the MVPA according to the SWA. This is comparable with Vassbakk- Brovold et al. [34] who also documented a higher proportion (i.e. 90%) of cancer patients perceiving themselves as meeting the MVPA recommendation of 150 min/week, while less than 50% actually met the PA recommendations recorded with SWA. This compares with a normal adult population in Norway, in which one in five met the national PA recommendations (i.e. 30 min/day) [21]. Importantly, the physical activity assessment method used in the normal Norwegian population survey was different from the one used in the present study and the study of Vassbakk-Brovold et al. [34]. Several barriers to meet PA recommendations among cancer survivors have been documented, of which treatment and disease-related factors are dominant [68, 69]. Consequently, cancer patients may feel breathlessness at lighter intensities than normal, as abovementioned, resulting in over-reporting of PA. Thus, these considerations are important to bear in mind when using self-reported data on PA in cancer patients.

The main strength in the present study was the use of SWA as the objective reference method in evaluating self-reported PA from the two questionnaires. Additionally, there was high compliance with the protocols for both self-reporting PA and wearing time of SWA. The NORDIET-FFQ and the HUNT-PAQ asked for PA in recent weeks (i.e. the previous 1–2 months), whereas the SWA recorded PA the subsequent week. Since none of the patients in the present study underwent chemotherapy during the validation period (i.e. mean time since last treatment of 155 days), less variation due to treatment effect on physical activity was therefore assumed. The limitation in our study was the use of different cut-off points defining frequency and duration of PA, which might have caused misclassification into MVPA, MPA and VPA activities between the questionnaires and SWA. This is supported by other studies, of which one should be aware of the different qualities in measuring levels of PA between methods [21, 66].

## Conclusions

There are many inherent limitations in using short questionnaires to assess PA as compared to objective monitors. In the present study we observed that the NORDIET-FFQ provided better specificity and better estimates of PA than the HUNT-PAQ in CRC patients. The HUNT-PAQ provided better sensitivity, and provided better ranking of PA and sedentary time among women than NORDIET-FFQ. However, it is important to be aware of the limitations when interpreting the results from these questionnaires. An objective monitor should be considered to be used when more accurate individual data on PA and sedentary time are needed.

## Additional files

Additional file 1: Physical activity, all participants in total and stratified by sex. Physical activity levels based on Norwegian Food Based Dietary Guideline (i.e. moderate intensity physical activity and vigorous intensity physical activity) are presented in minutes per week as both self-reported by NORDIET-FFQ and objectively measured by SenseWear Armband (SWA). (DOCX 18 kb)

Additional file 2: Bland-Altman plots depicting the mean differences (NORDIET-FFQ minus SenseWear Armband (SWA)) for physical activity in minutes per week; A. total-moderate intensity physical activity in minutes/week, B. total-vigorous intensity physical activity, minutes/week. The solid line represents the mean, and the dashed lines represent the 1.96 SDs of the observations. Females denoted as ♀ and males denoted as ♂. (TIF 1133 kb)

## Abbreviations

CRC: Colorectal cancer
FFQ: Food frequency questionnaire
HUNT-PAQ: The Nord-Trøndelag Health Study Physical activity questionnaire
ICD: International classification of diseases and related health problems
MET: Metabolic equivalent
MPA: Moderate intensity physical activity
MVPA: Moderate-to-vigorous intensity physical activity
NORDIET-FFQ: Norwegian dietary guidelines food frequency questionnaire
PA: Physical activity
SWA: SenseWear Armband Mini
TNM: Tumor node metastasis
VPA: Vigorous intensity physical activity
