# Supplementary material for: Validation of two short questionnaires assessing physical activity in colorectal cancer patients
Source: BMC Sports Sci Med Rehabil. 2018 May 29;10:8. doi: 10.1186/s13102-018-0096-2 (PMC5975662; doi:10.1186/s13102-018-0096-2)
Supplement: Supplementary file 1 — Physical activity, all participants in total and stratified by sex. Physical activity levels based on Norwegian Food Based Dietary Guideline (i.e. moderate intensity physical activity and vigorous intensity physical activity) are presented in minutes per week as both self-reported by NORDIET-FFQ and objectively measured by SenseWear Armband (SWA). (DOCX 18 kb) [file 13102_2018_96_MOESM1_ESM.docx]

**Additional file 1** Physical activity, all participants in total and stratified by sex

| Physical activity (min/week)^a^ | NORDIET-FFQ | | | SWA | | | NORDIET-FFQ/SWA  p-values^b^ | | |
| --- | --- | --- | --- | --- | --- | --- | --- | --- | --- |
|  | Total (n =78) | Men (n=42) | Women (n=36) | Total (n = 78) | Men (n=42) | Women (n=36) | p_tot_ | p_male_ | p_female_ |
|  | Median  (P_5_, P_95_) | Median  (P_5_, P_95_) | Median  (P_5_, P_95_) | Median  (P_5_, P_95_) | Median  (P_5_, P_95_) | Median  (P_5_, P_95_) |  |  |  |
| total-MPA | 152 (0, 469) | 159 (0, 469) | 152 (7, 469) | 629 (135, 1823) | 646 (133, 1519) | 607 (169, 1866) | <0.001 | <0.001 | 0.001 |
| total-VPA | 0 (0, 219) | 4 (0, 256) | 0 (0, 236) | 8 (0, 109) | 9 (0, 97) | 6 (0, 160) | 0.082 | 0.245 | 0.202 |

^a^Physical activity levels based on Norwegian Food Based Dietary Guidelines
^b^ Wilcoxon signed rank test, p-values for mean physical activity from NORDIET-FFQ and SWA, both total and between sex

NORDIET-FFQ, Norwegian Dietary Guidelines Food Frequency Questionnaire;SWA, SenseWear Armband; total-MPA= total moderate physical activity; total-VPA=total vigorous physical activity
