# Supplementary figures and images for: Validation of two short questionnaires assessing physical activity in colorectal cancer patients
Source: BMC Sports Sci Med Rehabil. 2018 May 29;10:8. doi: 10.1186/s13102-018-0096-2 (PMC5975662; doi:10.1186/s13102-018-0096-2)

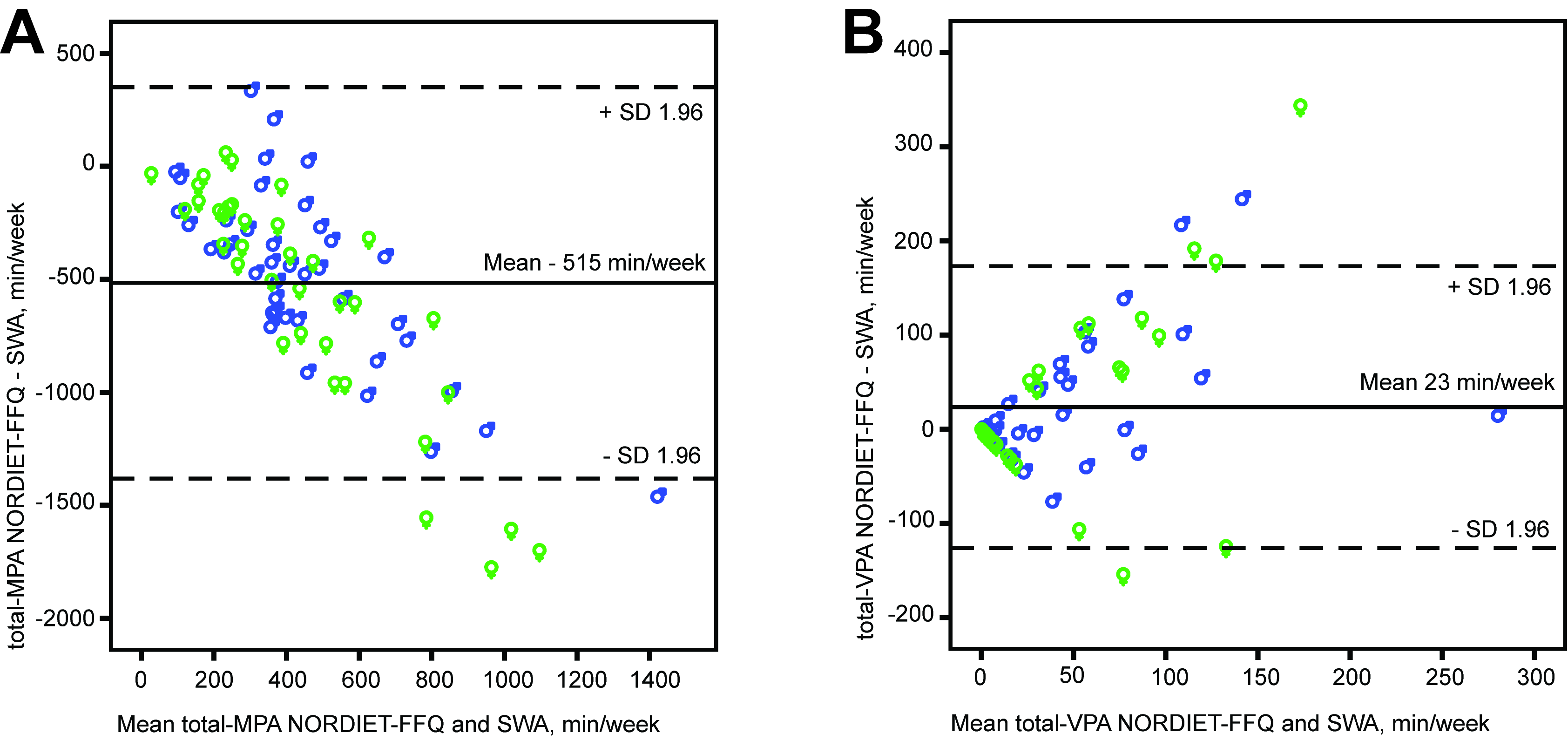

Supplement: Supplementary file 2 — Bland-Altman plots depicting the mean differences (NORDIET-FFQ minus SenseWear Armband (SWA)) for physical activity in minutes per week; A. total-moderate intensity physical activity in minutes/week, B. total-vigorous intensity physical activity, minutes/week. The solid line represents the mean, and the dashed lines represent the 1.96 SDs of the observations. Females denoted as ♀ and males denoted as ♂. (TIF 1133 kb) [file 13102_2018_96_MOESM2_ESM.tif]
